# Low prevalence of Human Papillomavirus in oral cavity carcinomas

**DOI:** 10.1186/1758-3284-2-6

**Published:** 2010-03-12

**Authors:** Jerry Machado, Patricia P Reis, Tong Zhang, Colleen Simpson, Wei Xu, Bayardo Perez-Ordonez, David P Goldstein, Dale H Brown, Ralph W Gilbert, Patrick J Gullane, Jonathan C Irish, Suzanne Kamel-Reid

**Affiliations:** 1 Department of Laboratory Medicine and Pathobiology, University of Toronto, Toronto, Ontario, Canada; 2 Division of Applied Molecular Oncology, Princess Margaret Hospital, Ontario Cancer Institute and University Health Network, Toronto, Ontario, Canada; 3 Department of Otolaryngology/Surgical Oncology, Princess Margaret Hospital, Ontario Cancer Institute and University Health Network, Toronto, Ontario, Canada; 4 Department of Biostatistics, Princess Margaret Hospital, University Health Network, Toronto, Ontario, Canada; 5 Department of Pathology, Toronto General Hospital, University Health Network, Toronto, Ontario, Canada; 6 Institute for Medical Sciences, University of Toronto, Toronto, Ontario, Canada; 7 Department of Medical Biophysics, University of Toronto, Toronto, Ontario, Canada

## Abstract

**Background:**

Increasing evidence shows that Human Papillomavirus (HPV) is preferentially associated with some head and neck squamous cell carcinomas (HNSCCs), with variable infection rates reported.

**Methods:**

We assessed HPV involvement in HNSCC using the Roche Linear Array HPV Genotyping Test, which can detect 37 different HPV types. We examined the prevalence of HPV infection in 92 HNSCCs (oropharynx, oral cavity, and other HNSCC sites).

**Results:**

HPV was frequently detected in oropharyngeal cancers (OPCs) (16/22, 73%), but was uncommon in oral cavity cancers (2/53, 4%), and in other HNSCC subsites (1/17, 6%). HPV positive tumors were associated with patients that were 40-60 years old (p = 0.02), and node positive (p = < 0.0001). HPV 16 was the most prevalent type, but other types detected included 6, 18, 33, 35, 45, and 52/58.

**Conclusion:**

Our results show that in contrast to oropharyngeal cancers, oral cancers and other HNSCCs infrequently harbor HPV.

## Introduction

In 2009, head and neck squamous cell carcinoma (HNSCC) had an estimated incidence of 48,010 cases (11,260 deaths) within the U.S. (American Cancer Society), and 4,550 cases (1,660 deaths) in Canada (Canadian Cancer Society). The most common risk factors for HNSCC development are excessive tobacco and/or alcohol consumption [1]. In addition, human papillomavirus (HPV) infection has been associated with some HNSCC subsites [2].

Most HPV research has primarily focused on cervical cancer, as >99% of cervical cancers harbor HPV [3]. Over 130 HPV types are known; these are classified as low- or high-risk based on their association with cervical carcinoma. HPV-16 and HPV-18 are the most commonly detected high-risk types [3]. High-risk HPVs promote tumorigenesis through expression of the E6 and E7 oncoproteins, which inactivate the tumor suppressors TP53 and RB1, respectively [4]. HPV-positive tumors often show CDKN2A/P16 over-expression due to a feedback mechanism involving RB1 inactivation [5].

Data from the literature show widely variable HPV infection rates in HNSCC [6-10]. This variability may be attributable to: sample storage and preparation of DNA [11]; ethnicity and geography [10,12]; small number of samples analyzed [10]; possible contamination [7]; detection technique used, and HNSCC site analyzed [6,7]. In a case-controlled study of HPV infection in HNSCC, HPV-16 was present in 72% of oropharyngeal carcinomas (OPCs), and associated with specific sexual behaviors [13], and marijuana use [14]. Unlike the striking association with oropharyngeal cancers, the incidence of HPV in other head and neck sites remains unclear. Interestingly, HPV positive HNSCCs are biologically different than HPV negative tumors, as shown by distinct gene expression profiles [15,16]. Importantly, patients with HPV positive HNSCC have better survival due to increased sensitivity to chemoradiotherapy [11].

We examined the prevalence of HPV in oral squamous cell carcinomas (OSCCs), compared to other head and neck subsites and OPCs. For HPV detection and genotyping, a sensitive and specific PCR-based method, the Roche Linear Array HPV Genotyping test was used for these studies.

## Materials and methods

### Patients

The University Health Network (UHN) Research Ethics Board approved this study; informed consent was obtained from all patients prior to sample collection. Medical records were examined to obtain detailed clinical and histopathological information, including age, sex, disease site, histopathological diagnosis, disease stage, history of tobacco and alcohol use, nodal metastasis, treatment, and outcome. Subsets of patients were classified as social or rare alcohol drinkers as written in their clinical reports. Patients having no use of tobacco or alcohol within one year prior to surgery were classified as former smokers and drinkers, respectively. Tumors were staged according to the current TNM classification, as recommended by the American Joint Committee on Cancer (UICC, 2002).

Patients over 60 years of age often present with HNSCC, whereas young patients with HNSCC are often characterized as patients under 35-45 years of age [17]. The patients examined in our study were stratified as young (≤ 40 years of age), intermediate age (>40 and <60) or older patients (≥ 60 years old). The median age was 59 (range, 22-93 years of age). The male-female ratio was approximately 2:1. Median follow up time was 22 months (range, 1-140 months).

### Tumor samples and DNA isolation

The 92 samples used in this study were retrospectively collected between 1995-2007. 78 HNSCC samples were obtained at the time of surgery from the Toronto General Hospital. Tissues were snap frozen and stored in liquid nitrogen. Also, 14 HNSCC were formalin fixed paraffin embedded samples. H&E stained sections were examined by histopathology (B.P-O.) to confirm >80% tumor in the specimens being tested.

DNA was isolated following fresh frozen tissue homogenization in liquid nitrogen using a cold steel mortar and pestle. Homogenized tissue was lysed in SNET buffer (1% SDS, 400 mM NaCl, 5 mM EDTA, 20 mM Tris, pH 8.0), containing 400 μg/mL proteinase K overnight at 55°C. After digestion, 25 mg/mL RNase was added and DNA was extracted by standard techniques using phenol/chloroform and ethanol precipitation. DNA quantity and quality was assessed by spectrophotometry (Nanodrop, Thermo Scientific, Waltham, MA) and electrophoresis on a 0.8% agarose gel. Genomic DNA was isolated from formalin fixed paraffin embedded (FFPE) samples using the DNeasy Blood and Tissue Kit (Qiagen, Valencia, CA).

### HPV detection

The Roche Linear Array HPV Genotyping test (Roche Diagnostics, Branchburg, NJ) was used for the detection of 37 low- and high-risk HPV types, according to manufacturer's instructions. Briefly, it utilizes biotinylated PCR of the HPV L1 region and reverse blotting to multiple HPV genotypes. HPV types were determined by lining up the manufacturer's HPV reference guide with the genotyping strip. A low- and high-copy β-globin internal control is included in each run to assess the quality of DNA sample. All experiments included an HPV positive control, an HPV negative control, and a no-template control. Cases that were HPV positive were repeated, without the presence of a positive control, to verify results and exclude the risk of contamination.

### Statistical methods

Descriptive statistics were examined as median and range for continuous variables, and frequencies and proportions for categorical variables. The Fisher's exact and Pearson's chi-square tests were used for statistical evaluation. Overall survival was calculated using the Kaplan-Meier method. A Cox Proportional Hazard regression model was applied for continuous predictors. Results were considered significant if p ≤ 0.05. Statistical analyses were performed using the SAS 9.1 software package (SAS Institute, Cary, NC).

## Results

All 92 HNSCC samples had a positive β-globin internal control on the linear array and were thus suitable for HPV analysis. Of these, 53 were from sites in the oral cavity: tongue, floor of mouth (FOM), palate, buccal mucosa and gingiva; 17 were from other sites (pharynx, nasopharynx, hypopharynx and larynx); and 22 were from the oropharynx (base of tongue and tonsil). The oropharyngeal cases were selected for comparison, as these tumors have a higher incidence of HPV infection.

**Figure 1:**
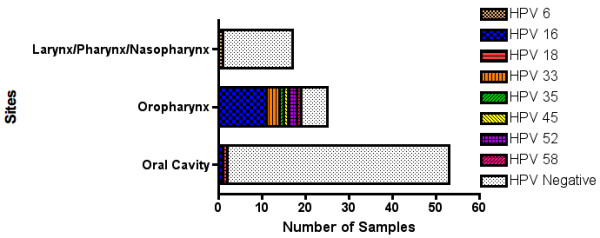
**Head and neck cancer sites and detected HPV types**. HPV types were highly associated with the oropharynx and rarely detected in oral cavity cancers and other head and neck cancer sites.

HPV positivity was stratified according to distinct HNSCC subsites: 2/53 (4%) OSCCs, 16/22 (73%) OPCs, and 1/17 (6%) tumors from other HNSCC sites were HPV positive. HPV positivity was significantly associated with OPCs (p < 0.0001) (Table 1). The most prevalent HPV subtype found in our analysis was the high-risk HPV-16 (12/19 cases) (Figure 1), which is the most common subtype observed in HNSCC [10]. We detected low-risk HPV-6 in one sample, and high-risk HPV types: 18, 33, 35, 45, and 52/58. 3/19 HPV positive cases, which were OPCs had confirmed multiple infections; and 1/19 HPV positive cases (Tongue SCC) had a low-level infection of HPV-18, as compared to the β-globin control.

**Table 1:** Statistical association of clinical factors and HPV status

Category	Variable	Patients (#)	HPV + (%)	p-value
**Age**	< = 40	22	1 (4.5)	0.02
	>40 and <60	28	10 (35.7)	
	> = 60	42	8 (19)	
				
**Sex**	F	28	4 (14.3)	0.32
	M	64	15 (23.4)	
				
**Smoking**	Y	53	11 (20.8)	0.59
	N	32	6 (18.8)	
	Former	5	0 (0)	
				
**Alcohol**	Y	46	9 (19.6)	0.47
	N	19	2 (10.5)	
	Former	6	0 (0)	
	Other	18	5 (27.8)	
				
**Stage**	I	6	0 (0)	0.12
	II	22	1 (4.5)	
	III	21	5 (23.8)	
	IV	40	10 (25)	
				
**Tumor Differentiation**	Well	1	0 (0)	0.51
	Moderately	68	12 (17.6)	
	Poorly	18	5 (27.8)	
	Other	5	2 (40)	
				
**Site**	Tongue	29	1 (3.4)	< 0.0001
	FOM	8	1 (12.5)	
	FOM+Tongue	6	0 (0)	
	Oropharynx (Base of Tongue)	22	16 (72.7)	
	Larynx/Pharynx/Nasopharynx	17	1 (5.9)	
	Other	10	0 (0)	
				
**Node**	+	43	15 (34.9)	< 0.0001
	-	46	1 (2.2)	
				
**Outcome**	Alive	83	17 (20.5)	0.24
	Dead	7	0 (0)	
				
**HPV**	+	19		
	-	73		

Clinical categories and the variables were examined for significant associations with HPV status. Statistical significance was assessed by Fisher's exact and Pearson's chi-square tests (p ≤ 0.05). M - Male; F -- Female; Y -- Yes; N -- No; FOM -- Floor of Mouth. Unknown values were not included in analysis.

HPV was significantly associated in node positive patients *vs. *node negative patients (p < 0.0001) (Table 1). When we stratified individuals into young (≤ 40 years old), intermediate age (>40 and <60 years old), and older (≥ 60 years old) HNSCC patients, HPV positive tumors were associated with the intermediate age patient group (p = 0.02). There were no significant associations between HPV and smoking or alcohol status. We also found no association between HPV infection and tumor differentiation and stage. In other studies, these associations have been inconsistent [6].

Survival was not significantly different for HPV-positive *vs. *HPV-negative HNSCCs (p = 0.24) (Table 1), as non-OPC sites were mainly HPV negative. Further examination of the 22 patients with OPC showed that outcomes for patients with HPV positive tumors included: 1/16 patients was alive with disease, 14/16 patients were alive with no evidence of disease, and 1/16 had lost to follow up. In contrast, 3/6 patients with HPV negative tumors were alive with disease, 2/6 patients were alive with no evidence of disease and 1/6 patients died of disease. Regardless of HPV status, we observed a significant gender-based difference in overall survival. Of the 7 deceased patients, 5 were females, non-smokers and non-drinkers; 3 of these were young and 2 were older patients; all of these patients were HPV negative. It has been suggested that a subgroup of young non-smoking female patients may have an aggressive form of HNSCC [18], although this would have to be tested in a larger cohort of patients.

## Discussion

In our study, we sought to determine HPV infection rates in OSCC, in comparison to other HNSCC sites such as OPC. We observed significant differences in HPV positivity among different HNSCC sites, with OPCs having the highest infection rate. We also detected a low infection rate within OSCC samples. In the literature, most HPV studies do not provide stratification between oral cavity subsites, e.g. mobile tongue *vs. *base of tongue; the latter is associated with a higher HPV prevalence due to the involvement of the oropharynx [11]. Indeed, high HPV prevalence has been consistently shown in OPC compared to a lower prevalence in other HNSCCs [19]. A recent comprehensive examination of the Surveillance, Epidemiology and End Results (SEER) database showed a significant increase in HPV-related *vs. *HPV-unrelated OPCs from 1973-2004 [20]. In this SEER analysis, HPV-related HNSCCs mainly involved the oropharynx (base of tongue, and palatine tonsils), whereas HPV-unrelated HNSCCs were from the mobile tongue, floor of mouth and palate. Gillison *et al. *used *in situ *hybridization for HPV detection and showed a higher frequency of HPV in tumors of the oropharynx, low frequency in larynx and oral cavity, and an absence of HPV in the hypopharynx or nasopharynx [14].

In our study, we observed a significant association of HPV positive tumors to be present in HNSCCs from intermediate age patients. It has been suggested that, from the time of sexual transmission of HPV, a clinical lesion can appear within two decades, in contrast to conventional risk factors (tobacco smoking and alcohol consumption), which may take several decades [21].

Interestingly, HPV positive HNSCCS have also been associated with lymph node metastasis. For example, in another study, HPV positive salivary samples were correlated with node-positive patients, suggesting that HPV infection may result from a cellular immunological deficiency that impedes the cancer cell's ability to clear HPV and thus result in cancer development, recurrence and metastasis [22]. Among patients with lymph node metastasis, HPV positive patients have a better prognosis than HPV negative patients, due to a better response to radiotherapy [23]. We did not detect significant differences in survival for patients with HPV positive *vs*. negative tumors, as most OSCCs were HPV negative in our study, and possibly due to the relative small sample size of OPCs analyzed.

Some studies have shown a higher prevalence of HPV in OSCCs and other head and neck sites outside the oropharynx. For example, examination of HPV status in OSCC from 50 Brazilian patients (excluding OPC), showed 24% HPV positivity, mostly HPV-16/18 [24]. In a Mexican patient cohort, a high frequency of HPV positivity (43.5%) was found in OSCC [25]. Such differences may be attributable to different HPV susceptibilities in different ethnicities/geographic regions or they may be due to different HPV detection methods. Boy *et al. *detected 7/59 (12%) HPV-18 positivity in oral cancer using quantitative real-time PCR and 0% positivity when using in situ hybridization assays on the same samples [26]. Our data have added to the literature by using a sensitive and comprehensive HPV detection and genotyping assay in HNSCC. This assay is currently used as a standardized diagnostic test for HPV detection and genotyping in cervical carcinomas in Europe [27]. In our study, by using this robust HPV detection and genotyping method, we showed that HPV is rarely involved in oral carcinomas and other non-OPC head and neck sites.

## Competing interests

The authors declare that they have no competing interests.

## Authors' contributions

JM was responsible for study design and content, literature research, experimental studies, data analysis, and manuscript preparation. PPR was involved in study design, data analysis, manuscript editing and review. TZ performed experimental studies. CS was responsible for data acquistion, and study concepts. WX perfomed the statistical analysis. BPO was involved with the data analysis. DPG, DHB, RWG, PJG, JCI were involved in study concepts, and sample acquistion. SKR was responsible for study content and design, manuscript editing and review.

All authors have read and approved the final manuscript.
